# Sleep Disturbances in Pediatric Craniopharyngioma: A Systematic Review

**DOI:** 10.3389/fneur.2022.876011

**Published:** 2022-05-13

**Authors:** Ramona Cordani, Marco Veneruso, Flavia Napoli, Natascia Di Iorgi, Claudia Milanaccio, Alessandro Consales, Nicola Disma, Elisa De Grandis, Mohamad Maghnie, Lino Nobili

**Affiliations:** ^1^Department of Neurosciences, Rehabilitation, Ophthalmology, Genetics, Maternal and Child Health (DINOGMI), University of Genoa, Genoa, Italy; ^2^Unit for Research & Innovation in Anesthesia, IRCCS Istituto Giannina Gaslini, Genoa, Italy; ^3^Department of Paediatrics, IRCCS Istituto Giannina Gaslini, Genoa, Italy; ^4^Neuro-Oncology Unit, IRCCS Istituto Giannina Gaslini, Genoa, Italy; ^5^Pediatric Neurosurgery Unit, IRCCS Istituto Giannina Gaslini, Genoa, Italy; ^6^Child Neuropsychiatry Unit, IRCCS Istituto Giannina Gaslini, Genoa, Italy

**Keywords:** craniopharyngioma, sleep, sleep-disordered breathing, excessive daytime sleepiness, narcolepsy, circadian rhythm, melatonin, hypocretin

## Abstract

Craniopharyngiomas are rare brain tumors of the sellar region and are the most common non-neuroepithelial intracerebral neoplasm in children. Despite a low-grade histologic classification, craniopharyngiomas can have a severe clinical course due to hypothalamic involvement. The hypothalamus plays a crucial role in regulating vital functions, and it is a critical component of the sleep-wake regulatory system. This systematic review aims to provide an overview of the current knowledge on sleep disorders in patients with craniopharyngioma to unravel their underlying mechanisms and identify possible therapeutic strategies. A comprehensive electronic literature search of the PubMed/MEDLINE and Scopus databases was conducted in accordance with the PRISMA^®^ statement. Extensively published, peer-reviewed articles involving patients with childhood craniopharyngioma and focused on this specific topic were considered eligible for inclusion. Thirty-two articles were included; a high prevalence of excessive daytime sleepiness was reported in CP patients, with wide variability (25–100%) depending on the diagnostic method of detection (25–43% by subjective measures, 50–100% by objective investigations). In particular, secondary narcolepsy was reported in 14–35%, sleep-disordered breathing in 4–46%. Moreover, sleep-wake rhythm dysregulation has been notified, although no prevalence data are available. Possible mechanisms underlying these disorders are discussed, including hypothalamic injury, damage to the suprachiasmatic nucleus, low melatonin levels, hypocretin deficiency, and hypothalamic obesity. The diagnosis and management of sleep disorders and associated comorbidities are challenging. This review summarizes the pathophysiology of sleep disorders in childhood-onset CP and the main treatment options. Finally, a possible diagnostic algorithm in order to accurately identify and treat sleep disorders in these patients is proposed.

## Introduction

### Craniopharyngioma: Epidemiology and Outcome

Craniopharyngioma (CP) is a rare embryonic brain tumor of the sellar and parasellar region that most likely arises from embryonic remnants of the craniopharyngeal duct epithelium, also known as Rathke pouch epithelium. CP may develop from the sella turcica upward to the third ventricle, affecting the hypothalamic-pituitary and the optic pathways regions (1, 2). Almost 50% of CP originate in the third ventricle floor, within the infundibulum and/or of the tuber cinereum, including the hypothalamus, spreading into the cavity of the third ventricle (1).

CPs account for 0.5–2.5 new cases per 1 million population per year, with 30–50% of all cases presenting during childhood and adolescence (1, 2). The adamantinomatous subtype occurs more frequently in younger patients (10 to 14 years of age), while the papillary subtype occurs more commonly in adult and elderly subjects (>50 years of age). CPs are the most common non-neuroepithelial intracerebral neoplasm in children (<18 years of age), counting 5–11% of intracranial tumors in this age group (1).

At the time of diagnosis, primary signs are frequently nonspecific manifestations of increased intracranial pressure (such as nausea and headache), visual impairment (losses of visual acuity and visual field) (62–84%), and endocrine deficits (52–87%) (2). In addition, growth impairment has been recognized in patients before diagnosis, while significant weight gain may occur over time (1).

Treatment for CP may include either radical surgical excision or subtotal resection followed by focal radiation therapy. Overall survival reported in pediatric cohorts ranges from 83% to 96% at 5 years, from 65 to 100% at 10 years, and 62% at 20 years (1). However, despite a low-grade histological classification (World Health Organization grade I) and high survival rates, quality of life is commonly impaired in long-term survivors due mainly to neuroendocrine comorbidities. Moreover, hearing and vision loss may occur (2).

The severe clinical course is mainly due to the hypothalamic-pituitary involvement by the tumor and/or possible treatment-related damage resulting in life-threatening sequelae (2, 3). Indeed, it should be considered that the hypothalamus plays a crucial role in regulating vital functions, such as the endocrine and autonomic systems and metabolic processes, and controlling hunger, thirst, thermoregulation and circadian rhythms (1, 4) and it represents a key component of the sleep-wake regulation system (5). Thus, hypothalamic damage by the tumor itself or by its treatment will result in hypothalamic disfunction and sleep disturbances (1–3, 6).

### Pathophysiology of Sleep Disorders in Craniopharyngioma

Sleep is a complex neurophysiological process that plays a key role in biological pathways essential to brain and body health. Sleep deprivation and sleep disturbances negatively impact several biological functions concerning the immune and autonomic nervous systems, inflammation, and metabolism (7, 8). Sleep disturbances can induce a change in autonomic control, hyperactivation of the orthosympathetic autonomic system, an increase in oxidative stress, an altered inflammatory response with an increase in inflammatory mediators, with a negative impact on endocrine-metabolic, cardiovascular, and immune functions. Nevertheless, a bidirectional connection has been described. Autonomic dysfunction and chronic autonomic hyperactivity may induce sleep disturbances (e.g., insomnia). Furthermore, sleep is impaired during activation of the immune system, and in endocrine-metabolic disorders (e.g., obesity, endocrine abnormalities) (7–11).

The timing and duration of sleep and wakefulness result from a complex and dynamic interaction between homeostatic and circadian processes (12, 13). The homeostatic drive grows with wake duration, indicating the increase in sleep need. The circadian process favors wakefulness in opposition to the homeostatic pressure to sleep, and it promotes sleep onset during the night-time hours. The circadian rhythm results from a complex network of organ clocks of which the suprachiasmatic nucleus of the hypothalamus is the principal regulator (13).

A close relationship between hypothalamic dysfunction and abnormal sleep is widely reported in several disorders of different origin. For example, narcolepsy type 1 is caused specifically by hypothalamic dysfunction, particularly by the destruction of hypocretin neurons, whose role is essential in maintaining wakefulness and inhibiting REM sleep, with possible differential modulatory effects on various subcomponents of the sleep phases (14). In addition, various diseases likely to result from hypothalamic damage, such as ROHHAD (rapid-onset obesity with hypothalamic dysfunction, hypoventilation, and autonomic dysregulation), Prader-Willi syndrome, and tumors involving the hypothalamic area can cause sleep disturbances (15–20). Sleepiness and sleep-related breathing disorders have been reported in association with pituitary hormone defects - such as TSH and ACTH – and with metabolic syndrome, all being commonly reported in craniopharyngioma patients; some of these sleep alterations may persist even after appropriate substantive therapy has been started (21, 22).

Thus, the genesis of sleep disturbances in hypothalamic dysfunction disorders is due to the role of the hypothalamus as a sleep regulator and its impact on endocrine-metabolic and autonomic processes, which, in turn, may influence sleep. Therefore, patients with CP may develop various sleep disturbances similar to other disorders with hypothalamic dysfunction, such as excessive daytime sleepiness, sleep-disordered breathing, and disturbances in circadian rhythm regulation. An extensive review of the literature was undertaken to provide an update of the current knowledge of sleep disturbances in patients with childhood-onset craniopharyngioma and to focus on the diagnostic workup and possible therapeutic interventions.

## Methods

This systematic review was conducted in accordance with the PRISMA^®^ statement (23).

### Literature Search Strategy

A comprehensive electronic literature search was conducted in PubMed/MEDLINE and Scopus databases in order to find relevant English-written published articles. The articles were identified using the following keywords: a) “craniopharyngioma” and b) “sleep,” “sleep disorders,” “sleep-related breathing disorders,” “sleep-disordered breathing,” “breathing disorders,” “apnea,” “excessive daytime sleepiness,” “hypersomnolence,” “narcolepsy,” “hypocretin,” “orexin,” “circadian rhythm,” “melatonin,” “stimulant.”

### Inclusion and Exclusion Criteria

Only in extenso published, peer-reviewed papers were considered eligible for inclusion. No limits were established in terms of publication dates. Review articles not focused on this specific topic and book chapters were excluded from this review.

Only reports and studies involving patients with pediatric-onset craniopharyngioma were considered eligible for inclusion. Studies involving only adult-onset patients were excluded.

**Figure 1:**
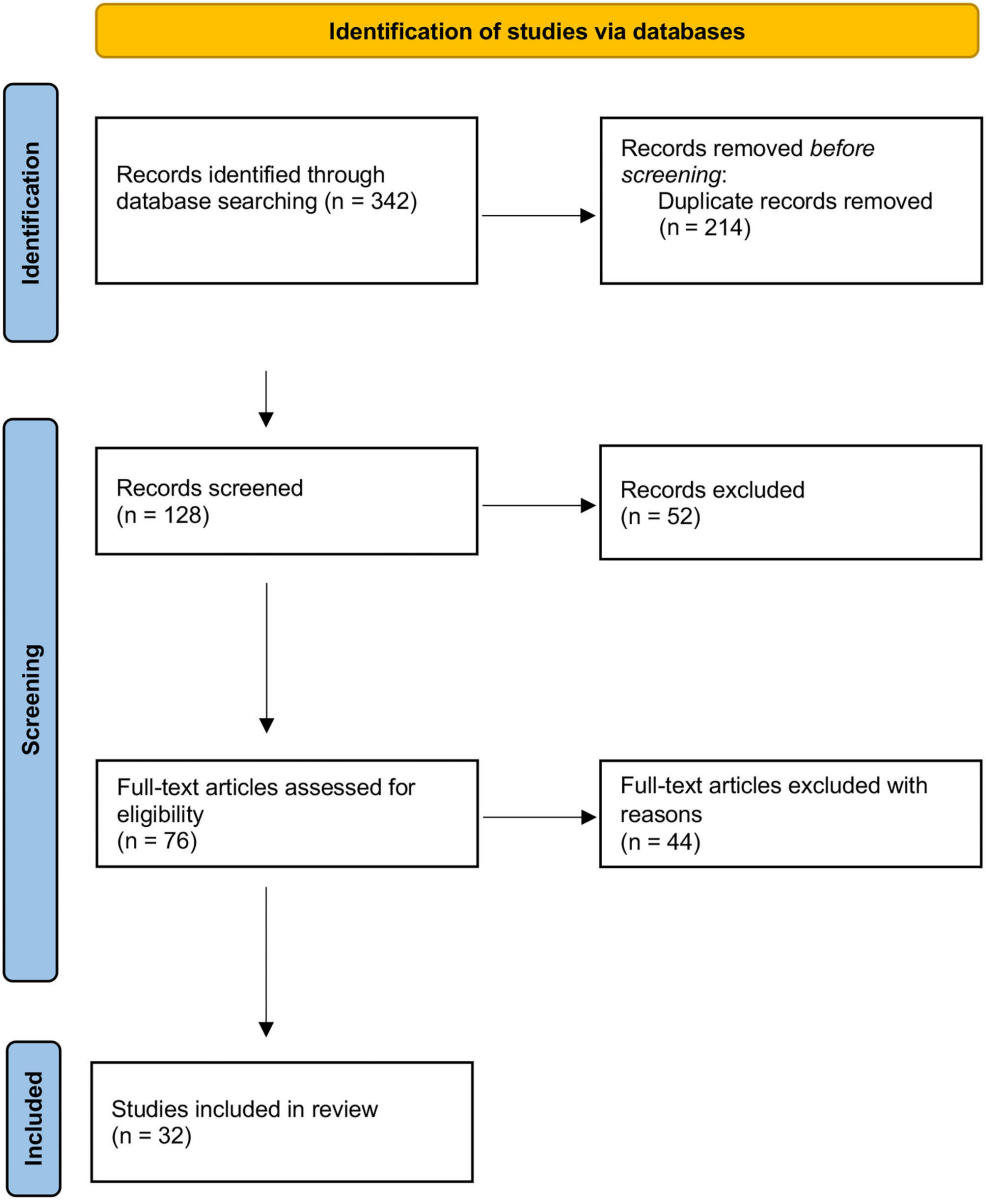
PRISMA Flow Diagram of search results. From Page et al. (23). For more information, visit: http://www.prisma-statement.org/

Two researchers (R.C., M.V.) independently reviewed the titles and abstracts of the retrieved articles, applying the inclusion and exclusion criteria. The two researchers then independently reviewed the full-text version of the remaining articles to determine their eligibility for inclusion (Figure 1).

### Data Extraction and Synthesis

The data extracted from all studies in line with our research objectives included participants characteristics (i.e., age of the subjects, age of tumor onset, clinical characteristics), procedures used evaluate sleep disorders (subjective and/or objective investigations), type of sleep disturbance, any additional examinations (melatonin dosage, CSF hypocretin), treatment and efficacy, if available.

## Results

The research identified 126 articles, of which 32 were included (Figure 1, Table 1). Study designs included observational retrospective and prospective studies, cross-sectional and case-control studies, case reports and case series.

**Table 1:** Main characteristics of the studies included in the review.

**References**	**Country**	**Design**	**Sample description**			**Sleep measures**	**Sleep disturbances**
			**Sample size** **(CP patients/total patients)**	**Age of patients** (yrs^*^)	**Childhood-onset (Childhood-onset CP patients/total CP patients)**		
Cordani et al. (24)	IT	Case series	2/2	19 and 12	2/2	Patient 1: ESS, PSG, MSLT, Actigraphy Patient 2: PSG	Patient 1: SDB, Narcolepsy Patient 2: SDB, parasomnias, *status dissociatus*
Crabtree et al. (25)	US	Cross-sectional	70/70	6 - 20	70/70	M-ESS MSLT PSG	28.8%: EDS by M-ESS 81.8%: EDS by MSLT 5.7%: SDB
Crowley et al. (26)	IRL	Case-control	28/28	19 - 67	7/28	ESS PSG	25%: EDS by ESS 46%: sleep apnea
Denzer et al. (27)	DE US	Case series	4/7	17	3/4	Clinical evaluation	1 patient: EDS, improvement with Dextroamphetamine
Honegger et al. (28)	DE	Cross-sectional	13/13	17-76	1/13	The Nottingham health profile for health-related quality of life	20%: pre-operative sleep problems (not specified) 8%: post-operative sleep problems (not specified)
Ismail et al. (29)	AUS	Case series	9/12	19.6 (median age in males); 15.1 (median age in females)	9/9	Clinical evaluation	8/12: EDS, improvement with dexamphetamine
Jacola et al. (30)	US	Cross-sectional	62/62	11 ± 4.0	62/62	PSG MSLT	76%: EDS
Kalapurakal et al. (31)	US	Cross sectional	25/25	1–15	25/25	Clinical examination	12%: sleep disorders (not specified)
Killeffer et al. (32)	US	Case report	1/1	5–10	1/1	Clinical examination	Disturbed sleep pattern (frequent falling asleep, reversal sleep rhythm)
Klages et al. (33)	US	Cross-sectional	84/84	10.27 ± 4.3	84/84	Actigraphy, PSG, MSLT	Significant correlation between hypothalamic tumor involvement and BMI and EDS
Lipton et al. (34)	US	Cross-sectional	3/3	15–22	3/3	Actigraphy	Nighttime activity, inappropriate daytime episodes of rest
Madan et al. (35)	US	Retrospective	3/10	6–16	3/3	PSG, MSLT	Narcolepsy type 2
Mandrell et al. (36)	US	Cross-sectional	98/98	3–20	98/98	PSG, MSLT	80%: EDS, 45%: hypersomnia due to medical condition 35%: narcolepsy 5%: OSA
Manley et al. (37)	US	Retrospective	28/28	10–32	28/28	Semi-structured patient interview and patient symptom report PSG	43% (12/28): EDS 43% (3/7): central/obstructive sleep apnea
Marcus et al. (18)	US	Case series	1/3	5	1/1	PSG, MSLT	Secondary narcolepsy
Müller et al. (38)	DE	Cross-sectional	79/79	3.5–33.2	79/79	ESS	35%: EDS (42% of severely obese) decreased nocturnal salivary melatonin levels
Müller et al. (39)	DE	Cross-sectional	79/79	6–33.2	79/79	ESS	EDS decreased nocturnal salivary melatonin levels 10/10: improvement after melatonin substitution
Niel et al. (40)	US	Cross-sectional	50/50	3–20	50/50	PSG Actigraphy	50%: hypersomnia
Niel et al. (41)	US	Cross-sectional	78/78	6–20	78/78	PSG, MSLT	82%: EDS
O' Gorman et al. (42)	US	Cross-sectional	15/15	10–21	15/15	PSG	SDB (OAHI higher than controls)
Palm et al. (43)	SE	Cross-sectional	10/10	7.1–22.9	10/10	Long-term EEG	Decreased rates of REM sleep, lower sleep efficiency
Pickering et al. (44)	DK	Case-control	15/15	18.2–70.2	4/15	Actigraphy, ESS	EDS reduced sleep time and efficiency low midnight melatonin
Pickering et al. (45)	DK	Case-control	7/7	20.6–68.5	1/7	PSG, MSLT	57%: hypersomnia
Poretti et al. (46)	CHE	Cross-sectional	21/21	<16	21/21	ESS	29%: EDS
Ramanbhavan et al. (47)	IND	Retrospective/ prospective	41/41	1–59	~ 50%	Questionnaire	15%: pre-operative sleep disorders (not specified)
Sakuta et al. (48)	JP	Case report	1/1	19	1/1	PSG MSLT	Secondary narcolepsy
Schultes et al. (49)	CHE	Case report	1/1	29	1/1	NA	OSAS (improvement after distal gastric bypass operation)
Snow et al. (50)	IL US	Cross-sectional	3/5	11–19	3/3	ESS, PSG, MSLT	EDS
Tachibana et al. (51)	JP	Case report	1/1	11	1/1	PSG, MSLT	Secondary narcolepsy
van der Klaauw et al. (52)	NL	Case-control	27/27	27–80	8/27	ESS	33%: EDS
Witcraft et al. (53)	US	Cross-sectional	80/80	2–20	80/80	Actigraphy	Poor sleep
Yang et al. (54)	CN	Cross-sectional	131/131	9–20	32/131	ESS	Worse EDS in bilateral-HI group

*IT, Italy; US, United States; IRL, Ireland; DE, Germany; AUS, Australia; SE, Sweden; DK, Denmark; CHE, Switzerland; IND, India; JP, Japan; IL, Israel; NL, Netherlands; CN, China. CP, craniopharyngioma; yrs, years; Y, yes; NA, not available; PSG, polysomnography; MSLT, multiple sleep latency test; ESS, Epworth Sleepiness Scale; M-ESS, Modified Epworth Sleepiness Scale; MEQ, Horne-Ostberg Morningness- Eveningness Questionnaire; EEG, electroencephalogram; SDB, sleep-disordered breathing; EDS, excessive daytime sleepiness; OSA, obstructive sleep apnea; OSAS, obstructive sleep apnea syndrome; BMI, body mass index; HI, hypothalamic injury.*

* *Age at the time of the sleep evaluation.*

*The majority of the included studies examined the occurrence of sleep disturbance only after treatment with a few exceptions appropriately reported*.

In the literature, sleep disturbances in CP survivors have been reported predominantly in case studies and small cohorts. The studies are heterogeneous in terms of sample size, tools used to evaluate sleep disturbances, and variability in the timeline of sleep evaluation. The majority of the included studies examined the occurrence of sleep disturbance only after surgical procedure of craniopharyngioma (with or without radiotherapy) with a few exceptions that have been appropriately reported below. Although included studies involved patients with childhood CP, sleep assessment was performed during adulthood in most cases. Due to the heterogeneity of the studies, meta-analysis was not possible.

Some studies investigating the onset of disturbances and the outcome of CP patients have reported the occurrence of sleep disturbances; however, without providing any assessment and characterization (28, 31, 47).

### Clinical Features and Etiology

#### Subjective and Objective Excessive Daytime Sleepiness

Excessive daytime sleepiness (EDS) in pediatric patients with craniopharyngioma had already being described in early reports. In fact, in 1970, Killeffer et al. described a young girl with CP and sleep pattern abnormalities after surgery, frequent falling asleep, reversal of day-to-night sleep rhythm (32). When analyzing in more detail the studies that investigate sleep disorders, EDS is recognized in 25–100% of cases, considering all studies regardless of the diagnostic technique used. The range of variability is likely affected by different sample sizes and different patient selection criteria. Different results are obtained by distinguishing between the diagnostic methods employed and whether the results identified by subjective and objective evaluations are considered separately. Lower prevalence values have been reported in studies investigating somnolence using questionnaires (25–43% of cases). Poretti et al. detected daytime sleepiness in nearly 29% of patients with childhood CP, using the German version of Epworth Sleepiness Scale (ESS) (46). Müller et al. investigating a sample of 79 patients with childhood CP using the ESS, reported daytime sleepiness in 42% of severely obese and in 35% of normal weight or less obese patients; hypersomnolence was documented in 33% of patients with CP by Van der Klaauw using the same questionnaires (52). The authors conducted a case-control study comparing healthy controls with patients treated for CP and patients with non-functioning pituitary macroadenomas (NFMA) and found that patients treated for CP or NFMA have increased daytime sleepiness despite normal sleep patterns (52). Manley et al. reported daytime sleepiness in 43% of patients who performed a semi-structured patient interview and patient symptom report (37). Crowley reported an EDS prevalence of 25% considering ESS values >10, although 71% of patients reported somnolence. In addition, the authors report no differences between pediatric and adult-onset CP patients (26).

On the other hand, the prevalence of EDS in CP patients appears to be higher (50-100% of cases) in studies conducted with an objective sleep assessment, including polysomnography (PSG) and Multiple Sleep Latency Test (MSLT), the gold standard for objective assessment of EDS. In a pediatric cohort, Crabtree et al. reported that 81.8% of participants had EDS, with a mean sleep latency (SL) value <10 min at the MSLT (25). The authors also reported that EDS was found in 28.8% of patients using a Modified ESS (M-ESS), thus demonstrating that patients often do not recognize or accurately report their sleepiness. Mandrell and colleagues found that 80% of patients with excessive daytime sleepiness out of 98 pediatric patients who completed PSG and MSLT after surgical resection of CP (36).

In 2017, Pickering and colleagues in a study involving 7 CP patients and 10 healthy controls assessed with sleep questionnaires, PSG, and MSLT, reported that patients felt sleepy more frequently than controls, 57% of patients had electrophysiological findings indicative of hypersomnia on MSLT (45). In a case control study, Snow et al. evaluated hypersomnolence in 5 children after resection of pituitary tumors, including 3 cases of CP. The authors observed an increase of both subjective (ESS total score 15.2 ± 2.8 in patients, 5.00 ± 2.00 in control subjects) and objective daytime sleepiness in all patients (mean MSLT sleep latency 10.3 ± 5.3 min in patients and 26.2 ± 1.1 min in control subjects; no data are provided depending on tumor type) (50). In 2019, using PSG and actigraphy, Niel et al. reported a prevalence of hypersomnia in 50% of young CP patients (age 3–20 years) (40). In 2021, Niel and colleagues reported EDS in 82% of 78 patients with CP assessed with MSLT (41). Jacola et al. performed a sleep evaluation with PSG, MSLT and modified version of the Epworth Sleepiness Scale (M-ESS) in 62 pediatric patients with CP, finding that 76% of the overall group met MSLT-based criteria for EDS. Patients with a more extensive hypothalamic involvement (HI) were more likely to meet objective, standardized criteria for EDS (30). Moreover, another study investigating the outcome of patients with CP and the relationship with hypothalamic injury (HI) patterns found that sleepiness assessed with the Epworth Sleepiness Scale was worse in bilateral-HI group than in mild-HI, unilateral-HI or no-HI groups (54). A recent study involving 84 youths confirmed a significant correlation between hypothalamic tumor involvement and body mass index (BMI) and daytime sleepiness (33).

#### Excessive Daytime Sleepiness and Secondary Narcolepsy

A potential disease responsible for marked sleepiness in patients with CP could be secondary narcolepsy. Of all the cases reporting increased sleepiness, only a few provide data on narcolepsy documenting a prevalence between 14 and 35% (33, 36, 40, 45). Mandrell et al. reported that overweight or obese patients were more likely to be diagnosed with hypersomnia or narcolepsy. Furthermore, at diagnosis, grade 2 hypothalamic involvement (anterior and posterior HI, including mammillary bodies) was associated with the diagnosis of narcolepsy (36).

Other reports on secondary narcolepsy in different disorders have revealed that it can occur in tumors involving the hypothalamic area, including craniopharyngioma. For example, Madan and colleagues who conducted a retrospective study on the characteristics and outcomes of secondary narcolepsy in 10 children, reported 3 patients with narcolepsy type 2 after treatment of CP (35). Marcus et al. who described the outcome of secondary narcolepsy in three children with brain tumors reported a 5-year-old girl with CP and narcolepsy without cataplexy. In this case, daytime sleepiness began 1 month before the diagnosis of craniopharyngioma (18). Patients with narcolepsy type 1 lack the influence of the wake-promoting neuropeptide hypocretin, resulting in impaired regulation of sleep and wake boundaries (flip-flop switch) (5). The current diagnostic criteria are a mean MSLT sleep latency (SL) <8 min, ≥2 sleep-onset REM periods (SOREMPs) on MSLT, and clear-cut cataplexy or cerebrospinal fluid (CSF) hypocretin−1 (hcrt-1) deficiency (55). Interestingly, a low level of hcrt-1 in the CSF [93 pg/ml and 70.8 pg/ml; cut-off value for narcolepsy: 110 pg/ml (56)] has been detected in CP patients suffering from narcolepsy with or without cataplexy suggesting the possibility that surgical tumor removal could lead to defective production of orexin and consequently induce daytime sleepiness (48, 51). In contrast, normal values of hypocretin in CSF were found in both studies by Snow et al. and Pickering and colleagues (45, 50). However, it should be noted that the patients described by Snow and colleagues had EDS but not narcolepsy (50). Similarly, Pickering et al. reported that the analysis was performed in a subset of patients, but not in the patient with electrophysiological criteria for narcolepsy (short mean sleep latency, 2/5 SOREMPs) (45). This sample included patients with adult-onset and pediatric-onset CP patients, but it is not specified whether CSF hypocretin was tested in the childhood-onset patient (Table 2).

**Table 2:** Clinical and neurophysiological features of patients with CP in whom CSF hypocretin assay was performed.

**References**	**Clinical data**				**PSG**	**MSLT**		**CSF Hypocretin**
	**Sample population (n. patients, age)**	**EDS**	**Cataplexy**	**Hypnagogic hallucination Sleep paralysis**	**%REM**	**MSL (min)**	**SOREMPs**	**(pg/mL)**
Sakuta et al. (48)	1, F, 19 yrs	Y	Y	N	38.1%	1.0	4/4 naps	70.8
Tachibana et al. (51)	1, F, 11 yrs	Y	N	N	40.9%	1.4	3/5 naps	93
Pickering et al. (45)	5, >18 yrs *	NA	N	N	NA	NA	<2/5 naps	423 (340–429)
Snow et al. (50)	4, <18 yrs **	Y	N	N	15.2 ± 4.3	10.3 ± 5.3	NA	133 ± 14 (124–154)

PSG, polysomnography; MSLT, multiple sleep latency test; EDS, excessive daytime sleepiness; SDB, sleep-disordered breathing; REM, rapid eye movement sleep; MSL, mean sleep latency; SOREMP, sleep onset rapid eye movement period; F, female; yrs, years; 0, no; 1, yes; NA, Not Available for subgroup of patients who performed CSF hypocretin dosage. ^*^The sample patients included both adult-onset and pediatric-onset CP patients, it is not specified whether CSF hypocretin has been tested in the pediatric-onset patient. **: symbol used as a pointer to an annotation.

#### Excessive Daytime Sleepiness and Sleep-Disordered Breathing

Another reasonable explanation for daytime sleepiness is the occurrence of sleep-disordered breathing (SDB). However, only a few of the studies reporting increased sleepiness have investigated the association with sleep-disordered breathing. Overall, the prevalence of sleep-disordered breathing seems to be between 4 and 46%. Manley et al. reported a 43% prevalence of central or obstructive sleep apnea in a total of 7 patients evaluated with PSG, compared to an original sample of 28 patients in whom 43% of cases reported increased sleepiness (31). On larger samples, the prevalence of sleep-related breathing disorders recorded is lower. Crabtree and colleagues reported a rate of SDB of 5.7% in a sample 68 patients of whom 81.8% had EDS on the MSLT (25). Similarly, other PSG studies have found a prevalence of sleep-disordered breathing (apnea-hypopnea index, AHI, ≥2 or 5) ranging between 4 to 5.8% (33, 36, 40). In a study that included both pediatric and adult-onset CP patients who were overweight or obese, the prevalence of obstructive sleep apnea reached 46%. In this study, BMI did not correlate with AHI, suggesting that obesity alone does not explain the prevalence of sleep apnea in patients with craniopharyngioma. Notably, subjects with craniopharyngioma were more somnolent than weight-matched control group subjects (71 vs. 17%, by ESS), and ESS was unrelated to AHI (26), suggesting the coexistence of other factors that favor an increased sleepiness. On the other hand, O'Gorman and colleagues who conducted a cross-sectional study of obese CP patients and obese controls showed that SDB (including both central and obstructive sleep apneas) was increased in CP patients than in BMI- matched controls, suggesting a direct role of the hypothalamus in the regulation of respiratory activity (42).

#### Circadian Rhythm Disorders

Considering the role played by the hypothalamus, it is possible to hypothesize that hypothalamic damage may lead to an alteration of the sleep-wake rhythm. Data in the literature indicate that patients with craniopharyngioma have increased rates of nighttime awakening, decreased sleep efficiency, and decreased rates of REM sleep compared to control subjects (34, 43). In studies that performed actigraphy recording, a trend toward greater fragmentation of day-night rhythmicity and irregular circadian function with irregular bedtime, increased sleep onset latency, wake after sleep onset, frequent nocturnal activity, inappropriate daytime rest episodes associated with a low mean 24-h plasma melatonin levels and decreased mean nocturnal melatonin levels have been reported (34, 53). Other studies have investigated the pattern of melatonin secretion in these patients, showing that decreased nocturnal melatonin levels are associated with increased daytime sleepiness (39, 44), decreased sleep time and sleep efficiency, sleep quality, and physical health (44). In particular, in one of these studies, three different patterns of melatonin profiles were observed: normal, absent midnight peak, and phase-shifted peak. Only patients with an absent midnight peak had impaired sleep quality, increased daytime sleepiness, and general and mental fatigue (44). The authors suggested that salivary melatonin may serve as a biomarker of circadian rhythm, and collection may help diagnose circadian sleep-wake rhythm disorders. Due to the potential influence of cortisol on sleep, saliva cortisol concentrations were evaluated in a few studies. While in one study, there were no detectable differences in daytime salivary cortisol concentrations when comparing patient group and controls (39), in another study, higher salivary cortisol concentrations were described, especially in the evening. In this study, high midnight cortisol was associated with an increased number of awakenings during the night and a trend toward a decrease in total sleep time (44).

#### Treatment

Regarding the treatment of sleep disorders, different therapeutic approaches have been used in the examined studies (Table 3). However, data on treatments are reported mainly in case reports and case series.

**Table 3:** Treatments of sleep disorders in patients with CP.

**References**	**Sample population**	**Disturbance**	**Treatment**	**Effect**
Müller et al. (39)	10, adult obese patients	EDS	Melatonin (6 mg)	Improvement (10/10 patients)
Crowley et al. (26)	5, adult overweight/obese patients	EDS	Modafinil	Improvement (4/5) (*one died before intervention)
Marcus et al. (18)	1, 5-year-old child	Secondary narcolepsy	Modafinil (200 mg) Methylphenidate (20 mg)	Improvement (No prolonged FU available)
Cordani et al. (24)	1, 19-years-old boy	Secondary narcolepsy	Pitolisant	Improvement
Ismail et al. (29)	12, obese adolescent/young adult patients (9 with CP)	EDS	Dexamphetamine (5 mg twice daily)	Improvement of EDS (8/12) Improvement of concentration and physical exercise tolerance (3/12) (1 discontinued for deteriorating health from tumor recurrence)
Denzer et al. (27)	1, 17-years old obese boy	EDS	Dextroamphetamine	Improvement of EDS
Crowley et al. (26)	7, adult overweight/obese patients	SDB EDS	NIV CPAP	Improvement of EDS
Cordani et al. (24)	1, 19-years-old boy	SDB EDS	NIV CPAP	Resolution of SDB, slight improvement of EDS
Snow et al. (50)	2, adolescent patients	SBD EDS	NIV CPAP	Resolution of SBD, no improvement of EDS
Manley et al. (37)	1	EDS	Correction of sleep hygiene	Improvement of EDS

*EDS, excessive daytime sleepiness; SDB, sleep-disordered breathing; NIV, non-invasive ventilation; CPAP, Continuous positive airway pressure. Drug dosages were included in the table when available in the articles*.

First, the benefit of melatonin treatment in patients with CP is reported. After reporting a decrease in nocturnal melatonin levels associated with an increase in daytime sleepiness, Müller et al. initiated an experimental substitution with regular daily doses of 6 mg melatonin in obese adult patients with childhood-onset craniopharyngioma demonstrating improvement in daytime sleepiness (reduction in ESS from > 10 prior to initiation of treatment to median ESS from 7-6 to 8- during treatment) (39).

Treatment with wake-promoting agents has shown beneficial effects in CP-related hypersomnolence and narcolepsy. Pharmacologic therapy for sleepiness in CP targets the dopaminergic (modafinil) (18, 26), dopaminergic and noradrenergic (methylphenidate) (18), or histaminergic (pitolisant) (24) pathways. The positive impact of stimulant drugs (dexamphetamine/dextroamphetamine) has been described on daytime sleepiness and on weight gain following hypothalamic damage (27, 29). It should be noted that a low benefit of stimulant therapy (dextroamphetamine, methylphenidate, modafinil, armodafinil) on sleepiness was reported in a retrospective study; however, the authors reported suboptimal follow-up of patients' symptoms (35).

Sleep-disordered breathing can be treated with non-invasive ventilatory support; however, among the studies evaluated, only a few report data on the treatment of sleep-related breathing disorders. Some authors have reported that CPAP therapy resulted in an improvement in daytime sleepiness (26). However, some authors have reported that non-invasive ventilatory support led to a complete resolution of sleep-disordered breathing, but no change in daytime somnolence (50) or a slight decrease with the persistence of pathological values (24). The benefits of other treatment approaches such as distal gastric bypass surgery on OSAS are also reported (49), however without data on sleepiness.

Finally, improvement in symptoms has been reported after correction of sleep hygiene by controlling behavioral and environmental factors that can interfere with sleep (37).

## Discussion

**Figure 2:**
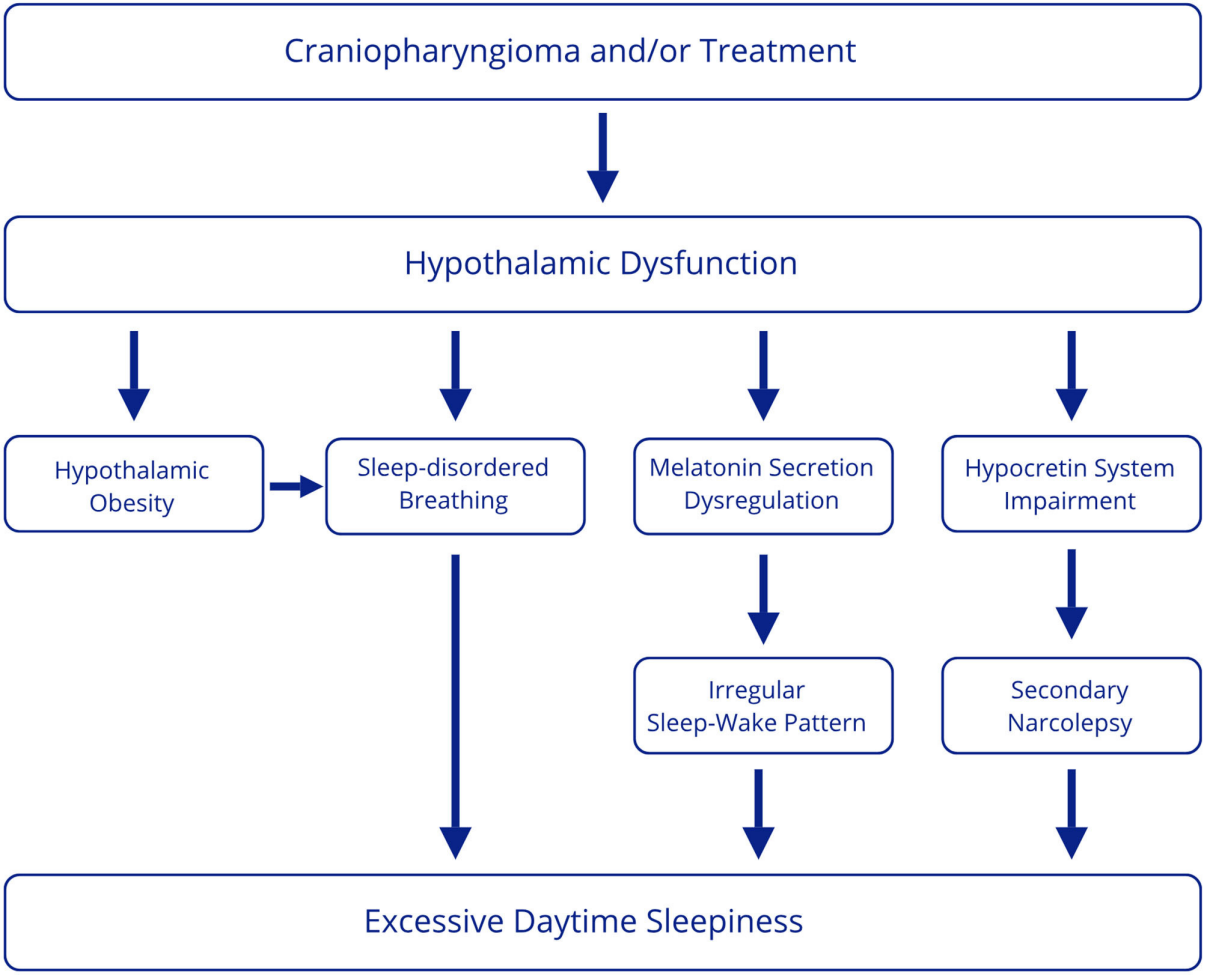
Multifactorial origin of excessive daytime sleepiness in CP patients.

This review aimed to examine sleep disorders in patients with craniopharyngioma. Data from the literature suggest that the most frequent disturbance is excessive daytime sleepiness, which appears to have a multifactorial origin. However, most studies do not assess a full diagnostic workup to identify associated sleep disorders. From the available data, the underlying conditions likely involved can be summarized as secondary narcolepsy, sleep-disordered breathing, and circadian rhythm dysregulation (Figure 2).

First, literature data demonstrate that secondary narcolepsy in patients with EDS after treatment for CP occurs at high rates (14–35%) (36, 40, 45), whereas the worldwide prevalence of narcolepsy in the general population is 25–50 persons per 100.000 (57). However, this rate may be underestimated, considering that not all patients with EDS undergo targeted diagnostic investigations. Narcolepsy is linked to the dysfunction of the hypocretin system in the dorsolateral hypothalamus, which projects rostrally to the forebrain and caudally to the tuberomammillary nucleus, nucleus accumbens, and raphe system, and regulates arousal, REM sleep, general sleep-wake balance, and stability of sleep stages (58). Notably, a low CSF level of hypocretin, the neuropeptide that promotes wakefulness and is typically reduced in patients with type 1 narcolepsy (57), has been detected in some CP patients with symptomatic narcolepsy (40, 48), suggesting the possibility that surgical removal of the hypothalamic tumor could cause defective orexin production and consequently induce narcolepsy and excessive daytime sleepiness. Low levels of hypocretin have also been observed in other disorders with hypothalamic dysfunction other than type 1 narcolepsy, such as ROHHAD (15). Moreover, lower hypocretin levels have also been reported in patients with Prader-Willi syndrome than in control subjects, but in some cases higher than in narcoleptic patients (56, 59–61). Secondary narcolepsy may be due to hypothalamic damage, which could be caused by tumor itself and/or tumor treatment. However, data on secondary narcolepsy are obtained from studies in patients after treatment, and no pre-surgical data are available. Therefore, it would be interesting to clarify the differences between patients who develop narcolepsy and those who do not by comparing tumor location and size and post-treatment hypothalamic damage, thus determining whether patients with involvement of specific areas develop narcolepsy. In addition, the interesting finding of low hypocretin levels obtained in case reports should be confirmed in larger samples of patients.

Another cause of excessive daytime sleepiness may be related to the occurrence of sleep-disordered breathing (SDB) reported with a frequency ranging from 4 to 46% according to different studies (25, 26, 36, 37, 40), thus more frequently than in the general population (1–5.8% of children, 10% of men and 3% of women aged 30 to 49 years) (62, 63). Patients with CP have various risk factors for SDB. First, patients undergoing hypothalamic surgery may exhibit abnormal food-seeking behavior and develop obesity and metabolic syndrome, that can lead to obstructive sleep apnea and hypoventilation syndrome (1). Moreover, a higher incidence of SDB has been reported in obese CP patients than in obese controls (42), suggesting that hypothalamic dysfunction may favor SDB through other mechanisms. Indeed, several hypothalamic areas are involved in the regulation of respiration and respiratory homeostasis, such as the paraventricular nucleus, perifornical area, dorsomedial hypothalamus, and lateral and posterior hypothalamus. In addition, the role of hypothalamic orexinergic neurons in modulating respiration has been reported (64) and the reduction of orexin neurons has been considered a possible mechanism favoring breathing disturbances in other disorders with hypothalamic dysfunction, such as Prader-Willi syndrome (59).

Despite the high incidence of SDB in CP patients, the relationship between obesity, SDB and EDS in CP is not so explicit. Indeed, EDS in patients with CP do not seem to be related to AHI and CP patients are more somnolent than controls with equal weight (26), suggesting that other factors may play a role in inducing sleepiness.

Finally, excessive daytime sleepiness could be secondary to circadian rhythm dysregulation. Alterations in the circadian rhythm have been shown in these patients. In particular, two studies, not included in this review as they did not involve pediatric-onset CP patients, showed alteration of 24-h body temperature (BcT°), a marker of circadian rhythm, in patients with CP before treatment with subsequent improvement during postoperative evaluation in most subjects (65, 66). Furthermore, chronotype alterations with a higher prevalence of the evening chronotype in CP patients have been reported (67).

As previously reported, the SCN is central for sleep regulation, being the primary endogenous circadian pacemaker. The blue light, through the retina and the retino-hypothalamic projection entrains the SCN which modulates the rhythm of diurnal cortisol and melatonin secretion. On the other hand, melatonin feeds back on the SCN to regulate its function. Therefore, melatonin promotes sleep, regulates circadian rhythms, and can be used as a marker of SCN function (44). Patients with CP may have impaired production of melatonin due to both hypothalamic and visual damage. However, the relationship between visual loss and sleep disorders was not sufficiently examined in the studies reviewed.

Alterations in melatonin production are reported in CP patients with disrupted circadian rhythm (34), excessive daytime sleepiness (38), impaired sleep quality, increased daytime sleepiness, and general and mental fatigue (44). In addition, improvement in daytime sleepiness has been reported after melatonin supplementation (39). Decreased EDS with melatonin treatment may indicate that impaired melatonin production and daytime circadian arousal mechanisms are responsible for increased daytime sleepiness and daytime naps. However, to demonstrate a causal association between impaired melatonin secretion, disturbed sleep-wake rhythm, and increased daytime sleepiness in these patients, it would be relevant to prove a decrease in sleepiness after correction of the sleep-wake rhythm disturbance (e.g., using sleep hygiene measures, melatonin) by performing objective sleep-wake rhythm analyses (actigraphy) and subjective and objective measures to assess excessive daytime sleepiness (e.g., questionnaires and MSLT).

In summary, the results of this review highlight a high prevalence of sleep disorders that, although heterogeneous, would all appear to result from hypothalamic dysfunction. In order to better clarify the pathogenetic mechanisms underlying the different disturbances, it would be helpful to carry out research aimed at examining sleep both before and after treatment to define the role of the tumor itself and the treatment and compare the type and severity of sleep disturbances with tumor position and surgery-induced injury.

The rate of sleep disturbances in CP patients is likely underestimated as not all survivors undergo a systematic sleep assessment. Moreover, CP patients may undervalue their disease or present atypical symptoms that are difficult to interpret, making the correct diagnosis even more challenging (24). Finally, since different sleep disorders can coexist in CP patients, it can be difficult to make a correct diagnosis and targeted intervention.

The diagnosis and treatment of sleep disorders is crucial, considering that sleep plays an essential role in overall health, cardiovascular and metabolic well-being, and immune system function (7, 8). In addition, sleep disturbances are associated with decreased cognitive abilities, increased anxiety and depression, and reduced perceived wellbeing (68).

**Figure 3:**
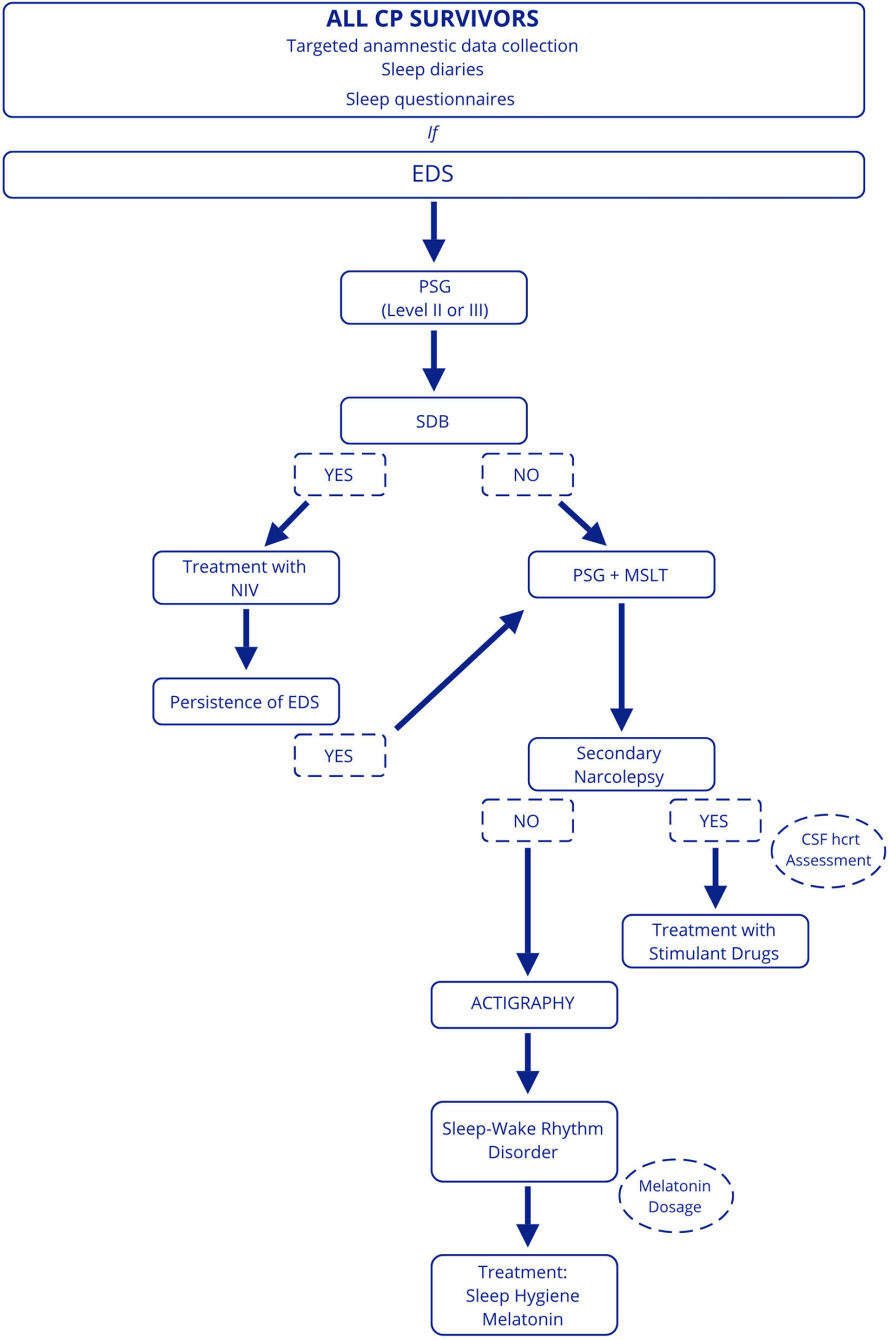
Proposed diagnostic work-up in patients with CP. EDS, excessive daytime sleepiness; PSG, polysomnography; SDB, sleep-disordered breathing; NIV, non-invasive ventilation; MSLT, Multiple Sleep Latency Test; hcrt, hypocretin.

For this reason, we believe that investigations aimed at identifying sleep disturbances are needed for all CP patients. A targeted anamnestic data collection, including sleep diaries and sleep questionnaires, should be performed in clinical practice to assess sleep quality and EDS, although data from the literature reveal that subjective assessment is not always reliable. In suspected sleep disturbances and EDS, patients should be referred to a sleep center. First, we suggest performing a level 2 or level 3 PSG to roll out the presence of SDB, considering the impact on cardiovascular and general health (63). If SDB is excluded, MSLT preceded by overnight PSG should be conducted to assess EDS and the presence of secondary narcolepsy. In patients with SDB, it will be appropriate in case of persistence of EDS after treatment with non-invasive ventilation to perform PSG and MSLT. Finally, actigraphy can be useful to evaluate the presence of sleep-wake rhythm disturbances in patients who have not been diagnosed with SDB and/or secondary narcolepsy or in those in whom EDS persists despite treatment and multiple concomitant disorders are suspected (Figure 3).

Concerning treatment, data from the literature show that sleep disturbances in these subjects require a multimodal approach. First, melatonin treatment, possibly by regulating the sleep-wake rhythm disturbance, has been shown to improve excessive daytime sleepiness. Second, stimulant therapies have positive effects in reducing sleepiness and treating secondary narcolepsy. Some drugs used in the treatment of narcolepsy have shown promising results in patients with craniopharyngioma with EDS or secondary narcolepsy, such as modafinil (18, 26), methylphenidate (18), dexamphetamine (29), and pitolisant (24). In particular, pitolisant, given its low cardiovascular risk profile, may be specifically indicated for CP patients, who have many metabolic comorbidities (69).

The optimization of hormonal replacement therapy and weight-loss interventions are also an important step to be considered in the multi-disciplinary approach to this challenging condition.

Finally, treatment with noninvasive ventilation has shown benefits on sleep-related breathing disorders, although rarely reduces daytime sleepiness (24, 26, 50).

## Conclusions

Sleep disturbances in patients with treated CP are common. They can have a multifactorial origin (including hypothalamic injury, damage to the SCN, low melatonin levels, hypocretin deficiency, hypothalamic obesity) and different clinical manifestations (EDS, secondary narcolepsy, SDB and Circadian Rhythm Sleep-Wake Disorders). These conditions can be difficult to diagnose and may lead to misdiagnosis and inappropriate treatment. However, proper treatment of these disorders can improve patients' quality of life and overall health.

## Data Availability Statement

The original contributions presented in the study are included in the article/supplementary material, further inquiries can be directed to the corresponding author/s.

## Author Contributions

RC, MV, MM, and LN: conception and design of the study, literature review and article selection, manuscript writing, and realization of figures and tables. FN, NDI, CM, AC, ND, and EDG: participation in the writing of the manuscript and drafting of figures and tables. All authors contributed to the article and approved the submitted version.

## Conflict of Interest

The authors declare that the research was conducted in the absence of any commercial or financial relationships that could be construed as a potential conflict of interest.

## Publisher's Note

All claims expressed in this article are solely those of the authors and do not necessarily represent those of their affiliated organizations, or those of the publisher, the editors and the reviewers. Any product that may be evaluated in this article, or claim that may be made by its manufacturer, is not guaranteed or endorsed by the publisher.
